# A little man of some importance

**DOI:** 10.1093/brain/awx270

**Published:** 2017-10-27

**Authors:** Marco Catani

**Affiliations:** NatBrainLab, Department of Neuroimaging and Department of Forensic and Neurodevelopmental Sciences, Sackler Institute for Translational Neurodevelopmental Sciences, Institute of Psychiatry, Psychology and Neuroscience, King’s College London, De Crespigny Park, London SE5 8AF, UK

## Abstract

Eighty years ago, Penfield and Boldrey introduced the homunculus in a paper published in *Brain*. In a reappraisal of the iconic aide-mémoire, Marco Catani reanalyses the original data, and argues that through its extended network the homunculus holds the key to the precise coding that results in coordinated activation of peripheral muscles.

The homunculus made his first appearance in the field of neurology on 1 December 1937, when Wilder Penfield and Edwin Boldrey (Fig. 1) published in *Brain* a 55-page article entitled *Somatic motor and sensory representation in the cerebral cortex of man as studied by electrical stimulation* (Penfield and Boldrey, 1937). The article is filled with painstakingly crafted summaries of data sourced from the cortical stimulation of 126 patients, who were operated under local anaesthesia by Penfield between 1928 and 1936. Compared to previous publications in animals, the authors had the advantage of operating on awake patients and relying on their verbal report of elicited movements and tactile sensation. All patient recordings were collated to obtain the first comprehensive map of motor and somatosensory localization in the human brain. This map was visualized as a distorted human-like figure—the homunculus—whose form indicates the amount of cortical area dedicated to motor or somatosensory functions of each body part.

**Figure 1:**
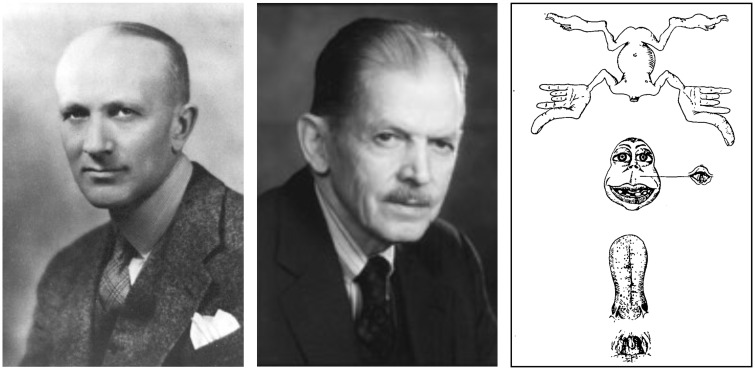
**Wilder Penfield (*left*) and Edwin Boldrey (*centre*), creators of the homunculus (*right*)**.

The paper had a long-lasting impact on both neurosurgical practice and scientific research. Clinically, it helped to establish direct cortical stimulation as an important method for functional mapping in neurosurgery; scientifically, it reinforced cortical localization as a valid paradigm to understand motor cognition, somatosensory perception and higher cognitive functions more generally. The homunculus was certainly their most striking proposition, but overshadowed other important conclusions made by the authors. First, while acknowledging the lack of histological analysis as a limitation of their study, they were confident they demonstrated the impossibility of confining ‘functional representation within strict cytoarchitectural boundaries’. Second, contrary to what others recorded in animals, the central sulcus of the human brain was not a clear boundary between motor and somatosensory areas. Many stimulations eliciting motor responses were indeed located in the somatosensory cortex of the postcentral gyrus and an even greater proportion of tactile responses in the motor cortex of the precentral gyrus. Surprisingly, the picture that emerged from their work was a clear degree of functional overlap between stimulation fields rather than an orderly sequence of segregated areas as their homunculus suggests.

For the 80th anniversary of this seminal paper, the authors’ original findings are re-examined with the intent to clarify the substrate that generated the homunculus, arguably the most reproduced yet unreplicated figure in neuroscience.

## What is the homunculus made of?

**Figure 2:**
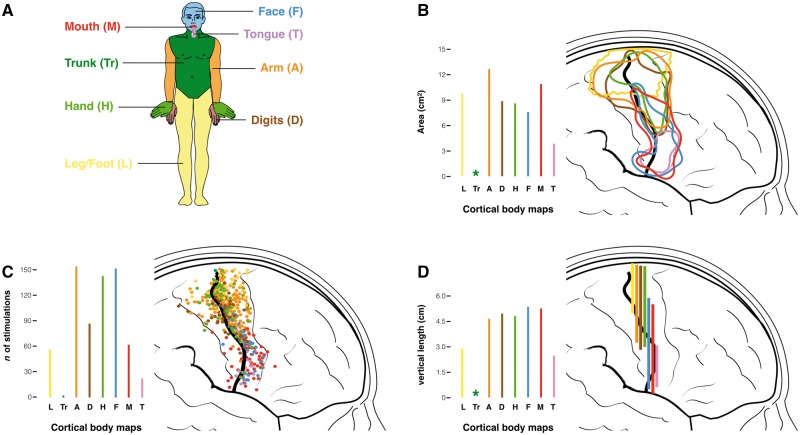
**Representation of the motor stimulations for different body parts.** (**A**) Colour-coding of different body parts. (**B**) Areas of the surface maps enclosing all motor stimulation points for each body part. (**C**) Count of the number of stimulations and (**D**) measurements of the vertical length of the surface maps for each body part. Original data are derived from Penfield and Boldrey (1937). The asterisk indicates the impossibility of generating any measurement of the area and length from the few stimulations recorded for the trunk. Similar maps for the somatosensory stimulations are provided in Supplementary Fig. 1.

In the Middle Ages, a homunculus was an artificial humanoid created through alembics and mysterious alchemy. In modern times, Penfield and Boldrey brewed their own version of the homunculus using more controlled, though no less controversial, ingredients. Their approach was systematic and laborious. The neurological homunculus was generated from 170 summary maps of the number and location of stimulation points for each body part, each patiently sketched by Boldrey from Penfield’s operation notes, photographs and drawings. The maps that displayed the positive responses for the toes, legs, trunk, arm, digits, hand, face, eyes, mouth and tongue were used to extract three separate measures for the motor (Fig. 2) and the somatosensory (Supplementary Fig. 1) stimulations: (i) the area for each body part as a 2D surface displayed on the lateral aspect of the central fronto-parietal cortex (Fig. 2B); (ii) the count of the total number of stimulations located in front and posterior to the central sulcus (Fig. 2C); and (iii) the vertical extent of each body area along the central sulcus (Fig. 2D).

In their attempt to visually summarize such a large amount of data, the authors faced challenges that are common to modern neuroimaging approaches (Friston *et al.*, 2004). First, the problem of quality control and data transformation, a step that in their case included elimination of artefactual stimulations (e.g. due to epileptic activity, shift in the proximity of the tumour, inability to replicate an evoked response, etc.) and spatial normalization to a common template. These steps were performed solely by visual inspection and manual transcription, processes that are vulnerable to error as evident from the several incongruences between the text and the figures in their paper. In the original text, for example, Penfield and Boldrey report a total of 21 motor stimulations for the mouth and lips (p. 405), which is inconsistent with both Fig. 7 indicating 64 motor points for the same body parts and Fig. 26 in which the stimulation points are more than 100.

The second challenge was represented by steps that included statistical transformation and inference. This step must have been particularly arduous in a predigital era and was omitted by the authors. This explains why their maps do not take into account variations in stimulus intensity, the number of subjects stimulated, the number of stimulations per subject, and the degree of overlap and density of the stimulations—all variables that could greatly affect the final results in many ways if not appropriately weighted. For example, the outer borders of those maps, and therefore their overall surface and the vertical extent along the central sulcus are influenced by the dispersion of the data, which in turn is affected by the number of outliers and measurements obtained from each subject. This may have led to a non-uniform overestimation or underestimation of body areas and influenced the final silhouette of the homunculus. Despite these limitations, their work remains an admirable attempt to capture the complexity of the interindividual variability, a pioneering effort that generated one of the most popular and iconic figures in the history of brain mapping, the motor-sensory homunculus.

## Back to the drawing board

In the original paper, the homunculus is nakedly portrayed as hanging upside-down with his head detached from the body and his pharynx and tongue extirpated from the mouth (Fig. 1). The corresponding figure legend explains that the homunculus ‘was prepared as a visualization of the order and comparative size of the parts of the body as they appear from above down upon the Rolandic cortex’. The terms ‘visualization’, ‘order’ and ‘comparative size’ deserve further scrutiny.

**Figure 3:**
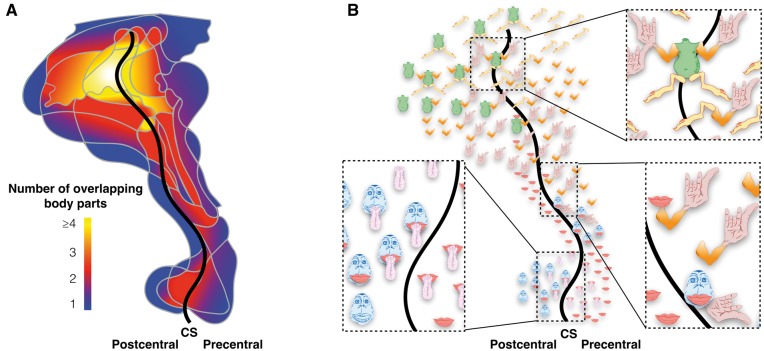
**Representation of the overlap between the somatosensory surface maps of different body parts.** (**A**) Heat map of the degree of overlap ranging from blue (one body part) to yellow (four or more body parts). (**B**) Map indicating the overlapping body parts of the homunculus. CS = central sulcus. Original data are derived from Penfield and Boldrey (1937).

Notwithstanding some of the methodological problems described previously, the visualization of the stimulation maps in an anthropomorphic form required omission of an important piece of information contained in the original maps: co-localization of stimulation sites for different body parts. This is clearly shown in Fig. 3, which represents a reanalysis of the original data for the somatosensory stimulations displayed as overlapping maps.

These maps indicate a high degree of overlay between different body parts, especially in the proximity of the central sulcus. Interestingly, the range of functional overlaps varies from expected—such as tongue and mouth, or arm and hand—to intriguing—such as mouth, arm and hand. While the limitations of their stimulation method and group analysis may have contributed to generating some of the overlaps (Farrell *et al.*, 2007), the frequent observation of co-stimulations in individual brains commonly reported by neurosurgeons during awake surgery (Foerster, 1936; Farrell *et al.*, 2007; Desmurget and Sirigu, 2015) suggests that this result should not be dismissed as solely artefactual. Indeed, using much more sophisticated microstimulation techniques with trains of longer duration, complex coordinated actions can be elicited in the animal brain (Graziano *et al.*, 2002). These have been interpreted as evidence of a functional aggregation of cortical output neurons dedicated to goal-directed synergic actions.

It may prove difficult to find a correspondence between co-activations recorded at the micro- and macroscopic level (Desmurget and Sirigu, 2015), but it is interesting to note that Penfield and Boldrey (1937) also reported complex motor responses for single stimulation sites. Some of these responses were characterized by bilateral ‘grimacing’ or ‘vocalization’, both of which require the coordinated activity of several muscles. They also noticed that the most common response for the digits was the movement of all the fingers together or combinations of two fingers that usually cooperate to produce a specific action, such as flexion of the index and thumb. Despite being aware of the possible teleological implications of the anatomical overlapping and co-activation, Penfield and Boldrey chose to highlight functional segregation. Forced to think out a way of displaying their findings they conceived the controversial homunculus (Schott, 1993).

Still used in contemporary teaching, the homunculus depicts two aspects of the organization of the pericentral cortex: the topographical order of body part representations and their altered proportions, reflecting the amount of cortex dedicated to a particular function. The motor cortical topography was already well established at the beginning of the 20th century in the brain of monkeys (Leyton and Sherrington, 1917) and humans (Foerster, 1936). Except for some marginal discrepancies, Penfield and Boldrey replicated previous findings in the sense that lower limbs are generally located above the areas of the upper arm, hand and fingers, and even more dorsal to the area of the face, mouth and tongue. However, their homunculus suggested clear-cut and orderly pattern that has rarely been replicated in single patients (Farrell *et al.*, 2007; Desmurget and Sirigu, 2015). In fact, quite often the movement or sensation of the same body part can be elicited by stimulations set widely apart, with other body parts occasionally interposed in between. These observations undermine the concept of a narrow functional localization of movement and somatosensory perception that the homunculus itself has greatly contributed to, and they are also at odds with the results of the precise topography documented in animals (Nelson *et al.*, 1980). Despite the obvious methodological differences, the well-documented observation of a functional recovery after extirpation or damage of specific cortical body areas suggests some caution in interpreting those maps as indicative of a one-to-one correspondence with a specific localized function. In clinical settings, it is probably fair to say that the homunculus provides only a very rough approximation of the likelihood of where the neurosurgeon might find activation of specific body parts; there is no doubt that Penfield and Boldrey were well aware of this. When commenting, for example, on the maps of the face, which is strangely in an upright orientation compared to the rest of the body, the authors admitted that a lack of sufficient stimulation points, of the nose, and the well-established location of the eye movements outside the face representation—in a region anterior to the hands/arms—may have generated a misleading representation.

Clearly, the homunculus cannot be taken at face value, but its most arresting feature of disproportionate body parts remains intriguing and largely valid to this day. With its long fingers, large mouth, and lumbering tongue, the homunculus has made a long-lasting impression. Its altered body proportions have been considered the expression of a basic physiological property of the pericentral cortex: the amount of pre- and postcentral cortex dedicated to a body part is not proportional to its size but to the degree of innervation that the cortex receives from or sends to that body part. Because body parts with highly skilled perceptual or motor functions receive a greater deal of neuronal innervation, the proportion of the homuncular body parts is generally thought to reflect specialization of function. This is quite obvious for the ability to discriminate two distant points, which is greater for those body parts that are enlarged in the corresponding cortical homunculus (Supplementary Fig. 2).

**Figure 4:**
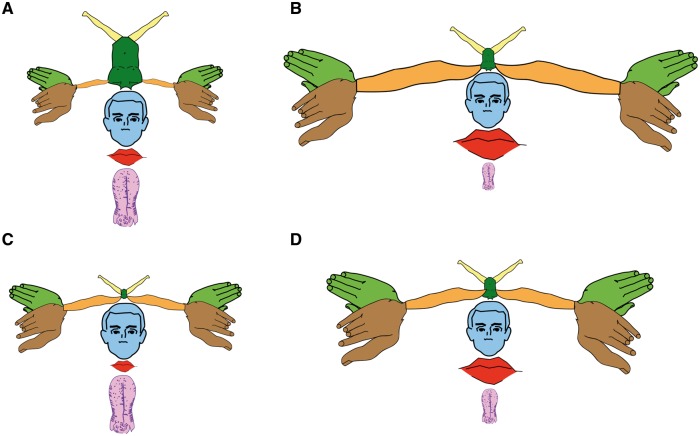
**The motor-sensory homunculus redrawn.** (**A**) The proportions of this first homunculus correspond to those of the original reproduced in Fig. 1. All other homunculi in **B–D** are derived from an average of the motor and somatosensory maps produced in Fig. 2 and Supplementary Fig. 1. (**B**) Homunculus generated from the surface maps. (**C**) Homunculus derived from the vertical length measurements. (**D**) Homunculus derived from the number of stimulation points. All measurements are from Penfield and Boldrey (1937).

This functional property of the cortex is certainly not exclusive to the sensory-motor neurons and one wonders why homunculus equivalents for the retinotopic representation of the eye (oculunculus?) or the tonotopic representation of the ear (aurinculus?) have not been put forward. Perhaps, Penfield and Boldrey were bold enough to propose something that they knew would have been considered a mere simplification by many others? After all, Leyton and Sherrington made a similar observation 20 years earlier in the brain of chimpanzees, orangutans and gorillas (Leyton and Sherrington, 1917). While Leyton and Sherrington used numbers to represent this property of the motor cortex (with each number corresponding to the stimulation of a specific group of muscles), Penfield and Boldrey made the controversial choice of using the vertical length along the pericentral cortex (Fig. 2D) to scale their homunculus. Somehow this information went missing when reproductions of the homunculus started to appear in popular textbooks, where its altered proportions were thought to reflect the extension of the area of each body part (Fig. 2B). To rectify this historical error, different versions of a modern motor-sensory homunculus have been generated using the data available in the original paper and presented in Fig. 4.

A comparison of the different homunculi shows that the original version was not proportionally scaled according to the measures reported in the 1937 paper. For example, the size of the tongue was clearly exaggerated in the first homunculus, a misrepresentation that Penfield remedied in a following publication (Penfield and Rasmussen, 1950). Also, when using the surface area, the representation of the arm is much larger and closer to that of the hand and fingers. This is perhaps not surprising as humans engage in many activities that require coordination of reaching (arm) and grasping (hand) for object manipulation.

While further homunculi can be generated from data presented in Fig. 2 and Supplementary Fig. 1, these diagrams are only of historical value for the impossibility of validation. Mapping the human homunculus using non-invasive techniques, such as transcranial magnetic stimulation or high field functional MRI, is an alternative but these approaches have their own limitations. Ultimately, the key to understanding the meaning of homunculus does not lie in the details of its figure but in the complexity of its connectional anatomy (Lemon, 1988).

## The legacy of the homunculus

It is difficult to guess what expectations Penfield and Boldrey had for their creation. In the original paper, the reader comes face-to-face with the homunculus after 42 pages and 27 figures, introduced with only a few lines of text and a single figure. Such modest presentation was followed by more than 10 years of silence from the authors, after which Penfield decided to replace the older homunculus with a new one, deemed to be of better proportions. But even for the second version of the homunculus Penfield did not have encouraging words: ‘[…] such drawings may easily become confusing if too much significance is attributed to the shape and comparative size’ (Penfield and Rasmussen, 1950). Still the homunculi multiplied in a rapid progression and were described in different regions of the human brain and in other species (Schott, 1993). The homunculus attracted also harsh criticism. Sir Francis Walshe, Consulting Physician to the National Hospital for Neurology and Neurosurgery, reading from his paper given at the Anglo-American Symposium in London stated: ‘[…] nor are the moderns content with maps, for homunculi and simiusculi have now made their horrid appearance, lineal descendants of Lewis Carroll’s Jabberwock, purporting to depict the fair face of nature, but in fact achieving something quite unnatural’ (Walshe, 1958)*.* Penfield himself was in the audience and it must have been painful to hear Walshe addressing his creature in those terms. I am not aware of any debate that might have taken place at the symposium but it is evident from Penfield’s publications prior and after this meeting that he never tried to promote his homunculus as the bearer of a new principle of brain organization. Instead, in his centrencephalic theory he proposed a central role of the thalamus in all brain functions while referring to the pericentral cortices as the ‘primary motor and sensory transmitting areas […] an arrival platform and a departure platform. Its function is to transmit and possibly transmute, with the aid of secondary motor areas, the patterned stream of impulses which arises in the centrencephalic system and passes on out to the target in voluntary muscles’*.* Clearly for Penfield the homunculus was not the puppeteer but simply the hand controller that allowed the thalamus to transmit its motor commands to the peripheral body. This was not a new concept as early researchers already recognized that brain stimulation activates not only the nearby neurons, but also an extended network of neurons sharing connections with those directly stimulated (Foerster, 1936). For this reason, brain stimulation has been seen as a method for probing network anatomy and function rather than cortical localization (Lemon, 1988).

The homuncular network has been recently studied with electrophysiological (Lemon, 1988; Davare *et al.*, 2011) and viral tracing studies (Rathelot and Strick, 2009) in animals and, more recently, with *in vivo* tractography in humans. The cortex of the homunculus is indeed highly and diversely connected to subcortical structures of the diencephalon, brainstem and spinal cord through projection pathways (Lemon, 1988). Direct and indirect pathways originate from pyramidal and non-pyramidal cells and project to spinal motor neurons directly or indirectly. Single motor neurons receive connections from multiple cortical neurons and single corticospinal axons project to several motor neurons. The final effect can be detected as stimulation or suppression of electromyographic activity. But the projection system originating from the motor cortex is not just an effector mechanism as it establishes reciprocal connections with its subcortical targets to enable a dynamic control of movements in action.

**Figure 5:**
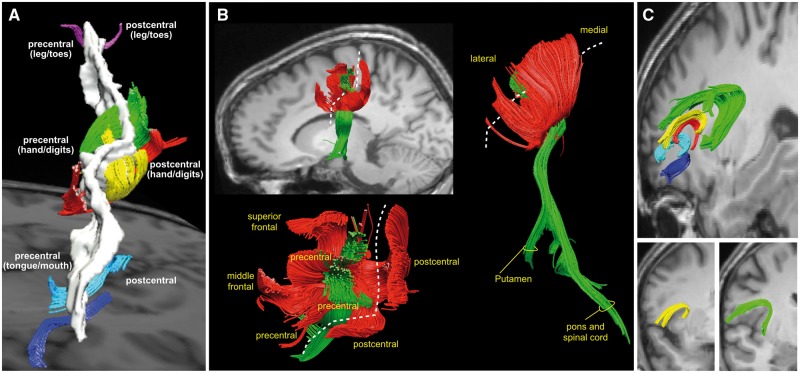
**Tractography-based reconstructions of large association and projection tracts of the homuncular cortex.** (**A**) Short association tracts connecting the precentral and postcentral gyri. In the hand knob region, these U-shaped tracts occupy a large volume and show a high degree of complexity (displayed in green, red and yellow colours). In the ventral region of the face and tongue (dark blue and cyan tracts) and dorsal region of the legs and toes (purple tracts) these connections are less prominent. (**B**) Short association (red) and long projection (green) tracts of the hand knob region from a lateral (*upper left*), dorsal (*lower left*) and posterior (*right*) view. The dashed line indicates the trajectory of the central sulcus. The short association tracts converge to the precentral regions of the hand knob area from the postcentral gyrus and the posterior regions of the superior and middle frontal gyri. The projection tracts are enclosed within the U-shaped tracts and connect the precentral gyrus to the putamen (corticostriatal fibres), the pontine nuclei (corticopontine tracts) and the spinal cord (corticospinal tract). (**C**) The fronto-insular tracts connect the frontal opercular cortex to the anterior insula. The connections from the precentral and subcentral/postcentral cortex are displayed in yellow and green, respectively. Please note that there is no correspondence between the colours used for these images and the colours in the previous figures. All images modified from Catani *et al.* (2012).

At the same time the motor cortex establishes reciprocal connections with other cortical areas of the frontal and parietal lobe through short association tracts (Fig. 5) (Catani *et al.*, 2012). It receives inputs from those anterior frontal areas forming a fronto-parietal system dedicated to motor planning for reaching and grasping movements (Davare *et al.*, 2011). It also receives direct inputs from the postcentral somatosensory cortex through a chain of U-shaped fibres located beneath the central sulcus. In the human brain these U-shaped fibres are particularly large in the hand region and progressively reduce in volume in the ventral region of the mouth and tongue and dorsomedial region of the leg and foot (Catani *et al.*, 2012). This anatomical pattern mirrors the homuncular maps derived from stimulation studies, and demonstrates a direct cross-talk between the motor and somatosensory homunculi. This communication across the central sulcus between the motor and somatosensory homunculus is important for learning and executing fine motor movements as indicated by ablation and stimulation studies in the monkey, as well as tractography studies in healthy humans and autistic patients with dyspraxia (Thompson *et al.*, 2017). In addition to reaching, grasping and speech, the homunculus attends to less electrifying tasks, such as mastication and vomiting. In these tasks the motor homunculus is assisted by the fronto-insular connections that convey visceral and sensory (especially gustatory and olfactory) inputs from the anterior insular to ventral motor regions that control orofacial movements (Catani *et al.*, 2012).

In conclusion, the considerations above place new emphasis on the need for studying the connectional anatomy of the pericentral cortex. A modern reappraisal of the homunculus should, therefore, consider it as the computational bottleneck within an extended network of cortico-cortical and cortico-subcortical connections dedicated to transforming cognition into action. As such, the homunculus holds the key to the precise coding that results in the coordinated activation of peripheral muscles. But the colloquial use of the term bears the risk of mistakenly granting the homunculus an existence in the realm of neuroscience: this little man, like many other figures that may naively populate our collective imagination, is just a metaphor for the complex neurological mechanisms that we strive to comprehend in their entirety. It gained popularity as a brilliant *aide-mémoire* and for this reason it will probably never lose its place in textbooks (Schott, 1993). For that, we owe Penfield and Boldrey a big hand.

## Supplementary Material

Supplementary Figures
