# Supplementary material for: A little man of some importance
Source: Brain. 2017 Oct 27;140(11):3055–61. doi: 10.1093/brain/awx270 (PMC5841206; doi:10.1093/brain/awx270)
Supplement: Supplementary Figures [file brain-2017-01620-file007_awx270.pdf]

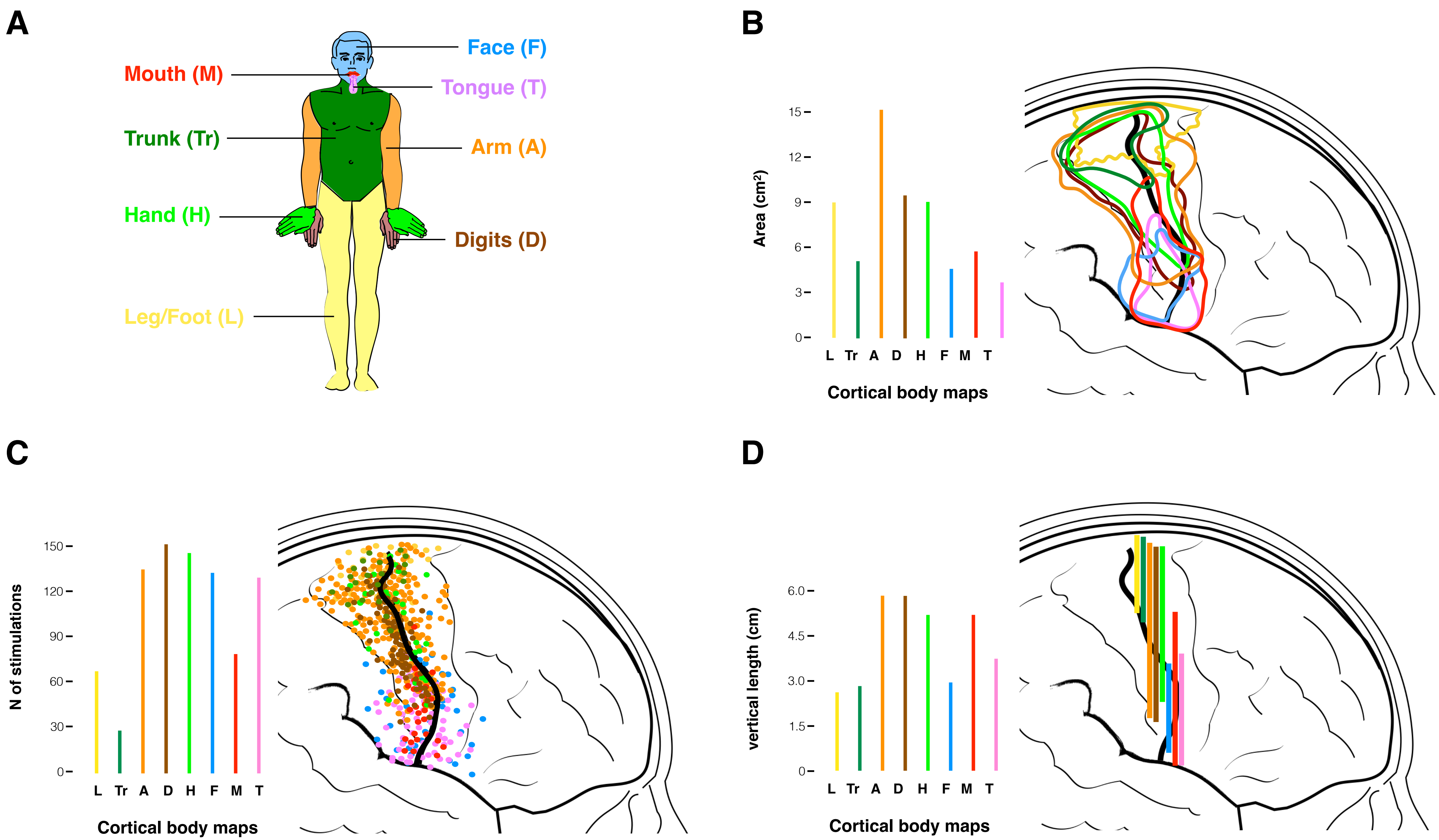


**Supplementary Figure 1.** Representation of the somatosensory stimulations for different body parts (according to the colour-coding shown in A). B) Measurements of the area of the surface maps enclosing the somatosensory stimulations of each body part. C) Count of the number of stimulations and D) measurements of the vertical length of the surface maps. Original data are derived from Penfield and Boldrey 1937.

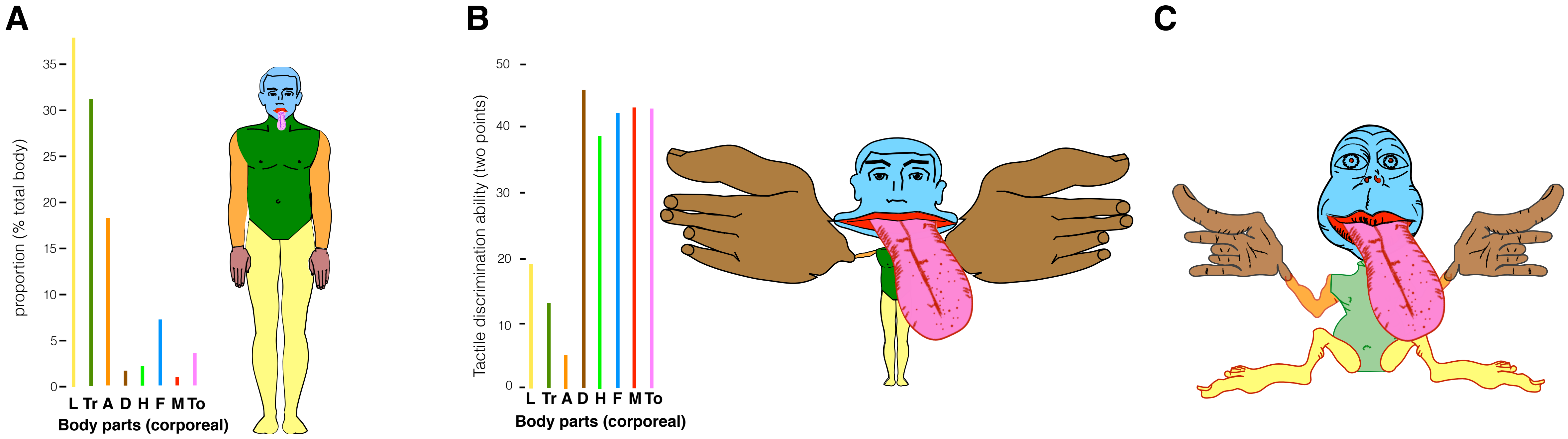


**Supplementary Figure 2.** Representation of the proportion of different body parts based on the measurement of the A) human body surface and B) tactile discrimination ability. C) The original homunculus of Penfield and Boldrey (1937) re-coloured. The measurements in A) are calculated as percentage of the total surface of the average human body. The measurements in B) are calculated according to the following formula: 50-distance between two points detected (higher values indicate better tactile discrimination ability).

**Supplementary Material - *Methods used for creating the images in Figure 2, 4 and Supplementary Figure 1.***

Figure 2 and Supplementary Figure 1 supplementary were drawn using the original material contained in the original paper by Penfield and Boldrey (1937) and reproduced in the next page. In particular, the measurements for the histogram of Figure 2C and Supplementary Figure 2C were taken from figures 26 and 27 in the original article. For the leg/ foot, the number of stimulations of the toes, ankle, knee, and hip were pooled together. For the arm, the number of motor stimulations of the shoulder, elbow, wrist were pooled together. For the leg/foot, the number of tactile stimulations of the shoulder, arm, elbow, forearm, wrist were used. The brow, eyelid, and jaw were used for calculating the motor stimulations of the face; the eye, nose, face, jaw and teeth for the tactile stimulation of the face. It is important to note that in figure 26 all face movements are included under the heading of "lips", which tends to give an exaggerated estimate of the real number of stimulations of the mouth and lips. Furthermore this is in contrast with what was reported in the original text (page 405) and in the corresponding figure 7. For this reason, the value for the motor stimulation of the tongue was calculated directly from original Figure 7.

Images from Figure 4 were created using average values of measurements presented in Figures 2 and Supplementary Figure 1. The proportions of each body part were rescaled according to the length except for the mouth where width was used instead.

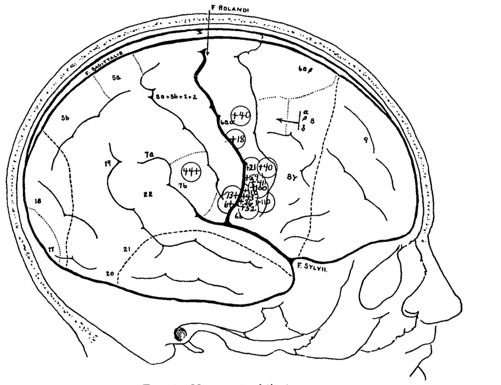

Fig. 5.—Movements of the tongue.

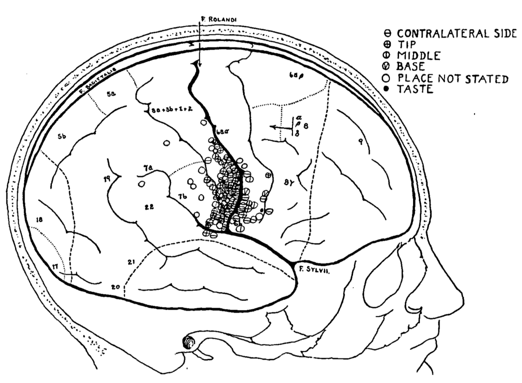


Fig. 6.—Sensation in the tongue.

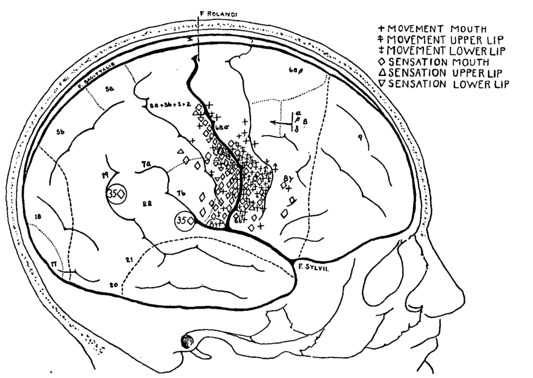


Fig. 7.—Movement and sensation of the mouth.

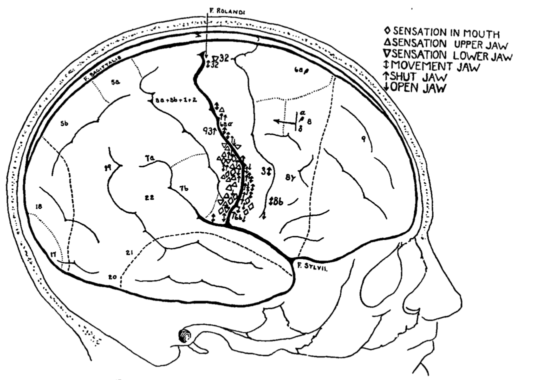


Fig. 8.—Jaw movement and sensation within the mouth.

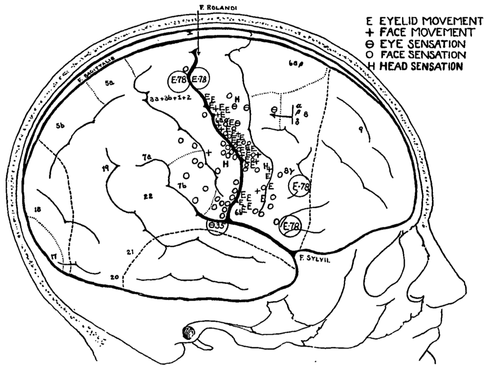


Fig. 9.—Movement and sensation of the face.

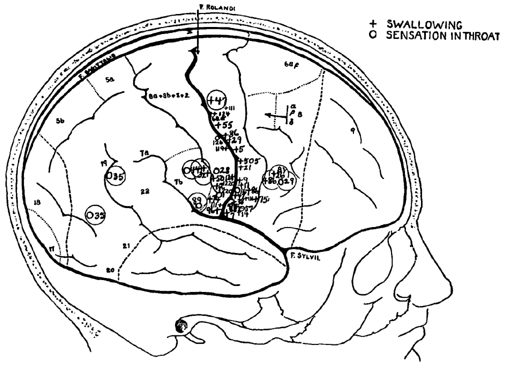


Fig. 10.—Swallowing and throat sensation.

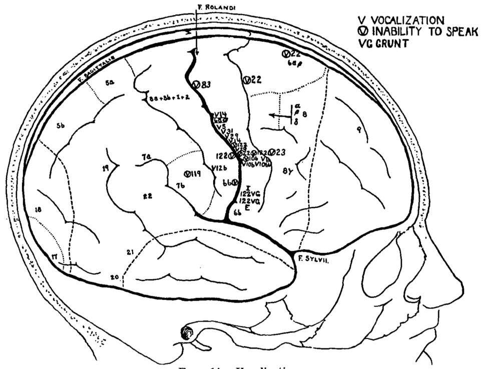


Fig. 11.—Vocalization.

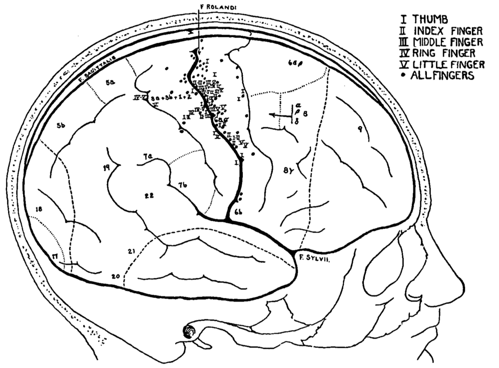


Fig. 12.—Movement of digits.

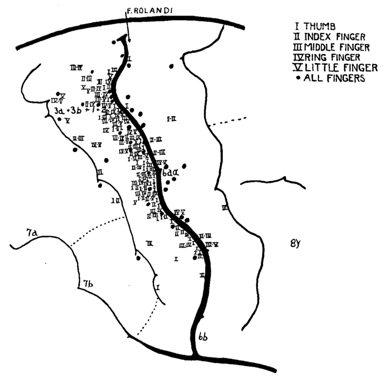


Fig. 13.—Sensation in digits.

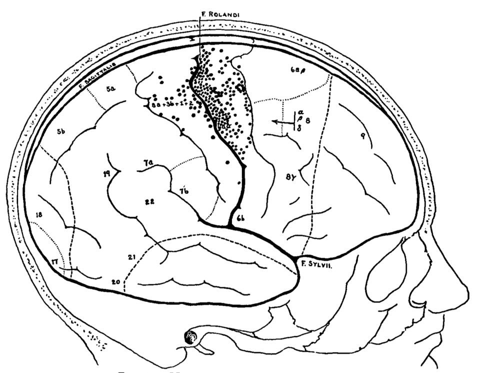

Fig. 14.—Movement of hand, arm and shoulder.

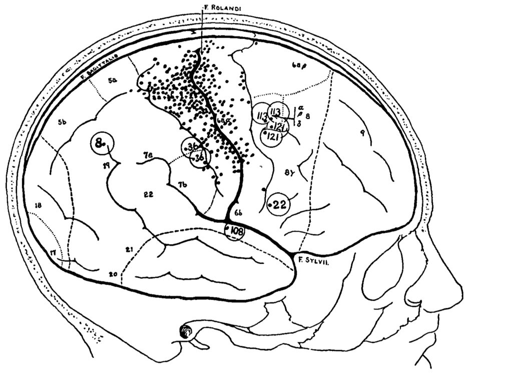

Fig. 15.—Sensation of hand, arm and shoulder.

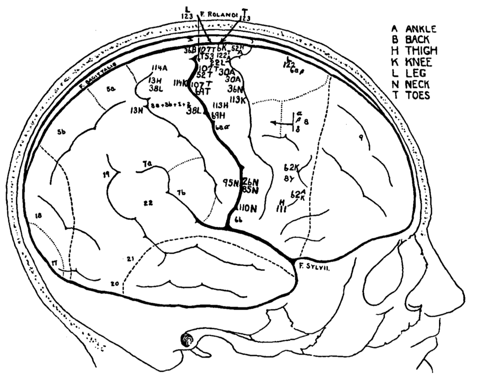


Fig. 16.—Movement of trunk and legs.

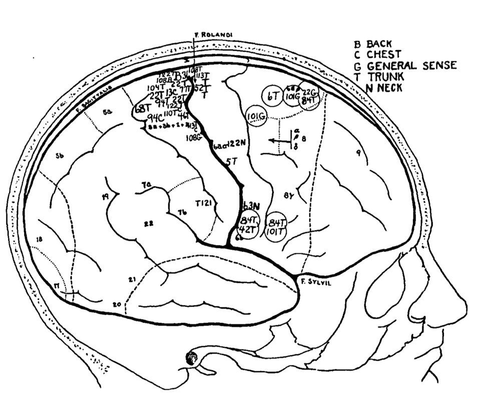


Fig. 17.—Sensation in trunk.

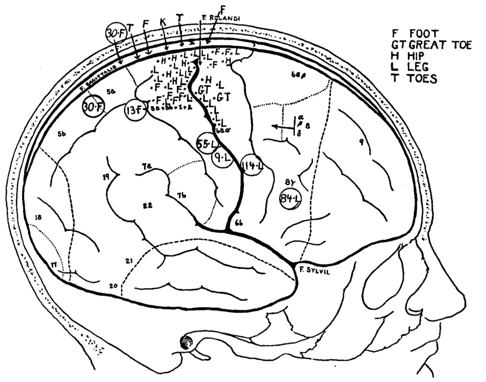


Fig. 18.—Sensation in leg and foot.

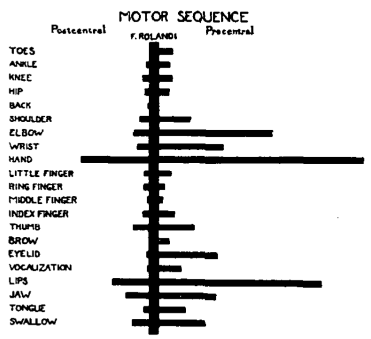


Fig. 26.

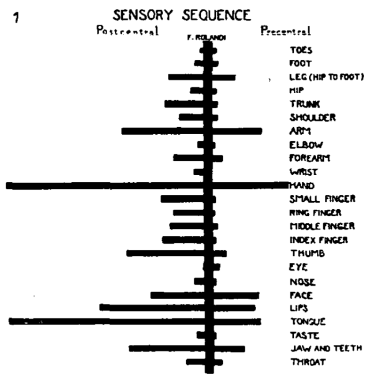


Fig. 27.
